# The relationship between hippocampal changes in healthy aging and Alzheimer’s disease: a systematic literature review

**DOI:** 10.3389/fnagi.2024.1390574

**Published:** 2024-08-15

**Authors:** Michael Woodward, David A. Bennett, Tatjana Rundek, George Perry, Tomasz Rudka

**Affiliations:** ^1^Austin Health, University of Melbourne, Heidelberg, VIC, Australia; ^2^Rush Alzheimer’s Disease Center, Rush University Medical Center, Chicago, IL, United States; ^3^Evelyn F. McKnight Brain Institute, Department of Neurology, Miller School of Medicine, University of Miami, Miami, FL, United States; ^4^Department of Neuroscience, Developmental and Regenerative Biology, University of Texas at San Antonio, San Antonio, TX, United States; ^5^Danone Specialised Nutrition, Hoofddorp, Netherlands

**Keywords:** Alzheimer’s disease, cognitive aging, hippocampal atrophy, hippocampal volume, mild cognitive impairment, systematic literature review

## Abstract

**Introduction:**

Neurobiological changes in the hippocampus are a common consequence of aging. However, there are differences in the rate of decline and overall volume loss in people with no cognitive impairment compared to those with mild cognitive impairment (MCI) and Alzheimer’s disease (AD). This systematic literature review was conducted to determine the relationship between hippocampal atrophy and changes in hippocampal volume in the non-cognitively impaired brain and those with MCI or AD.

**Methods:**

This systematic review was guided by the Preferred Reporting Items for Systematic Reviews and Meta-Analyses (PRISMA) methodology. The PubMed database was searched up to September 15, 2022, for longitudinal magnetic resonance imaging studies reporting hippocampal atrophy or volume change in cognitively normal aging individuals and patients with MCI and/or AD. Study selection was divided into two steps: (1) identification and retrieval of relevant studies; (2) screening the studies by (a) title/abstract and (b) full text. Two teams, each consisting of two independent reviewers, determined whether the publications met the inclusion criteria for the systematic review. An evidence table was populated with data extracted from eligible publications and inclusion in the final systematic review was confirmed.

**Results:**

The systematic search identified 357 publications that were initially screened by title/abstract, of which, 115 publications were retrieved and reviewed by full text for eligibility. Seventeen publications met the eligibility criteria; however, during data extraction, two studies were determined to not meet the inclusion criteria and were excluded. The remaining 15 studies were included in the systematic review. Overall, the results of these studies demonstrated that the hippocampus and hippocampal subfields change over time, with both decreased hippocampal volume and increased rate of hippocampal atrophy observed. Hippocampal changes in AD were observed to be greater than hippocampal changes in MCI, and changes in MCI were observed to be greater than those in normal aging populations.

**Conclusion:**

Published literature suggests that the rate of hippocampal decline and extent of loss is on a continuum that begins in people without cognitive impairment and continues to MCI and AD, and that differences between no cognitive impairment, MCI, and AD are quantitative rather than qualitative.

## Introduction

Progression of the “Alzheimer’s disease (AD) continuum” is not fully understood, with many individuals transitioning from age-related memory decline to increasingly severe cognitive impairment over a period of several years. One study estimated that by 65 years of age, the annual probabilities of transitioning to a state of more severe cognitive impairment were 8% for individuals with normal cognition, 22% for people with mild cognitive impairment (MCI) due to AD, 25% for patients with mild AD, 36% for patients with moderate AD, and 16% for patients with severe AD; with the likelihood of progression increasing with age for each cognitive state (Davis et al., 2018).

It is generally accepted that brain changes begin many years prior to the clinical manifestations of AD and that the spectrum of AD can span from clinically asymptomatic to severely impaired (Aisen et al., 2017). For example, a recent cohort study found that at any age, biologically-defined AD (i.e., using biofluid biomarkers: A+, T+) is more common than clinically-diagnosed probable AD (i.e., using conventional definitions based on clinical symptoms) due to many asymptomatic individuals with biological AD (Schneider et al., 2009; Jack et al., 2019). In addition, data suggest that among individuals without cognitive impairment, a smaller hippocampal volume is associated with cognitive decline (Fleischman et al., 2013).

It is well established that hippocampal volume decreases and hippocampal atrophy increases prior to the development of cognitive impairment (e.g., Fjell and Walhovd, 2010; Fraser et al., 2015; Nobis et al., 2019) and clinical dementia (e.g., Barnes et al., 2009; Peng et al., 2015). Research demonstrates that hippocampal volume begins to decline in midlife (Vinke et al., 2018; Chauveau et al., 2021), with an annual loss of approximately 1.18% in individuals over the age of 50 (Raz et al., 2004). Likewise, the rate of hippocampal atrophy also accelerates with increasing age (Vinke et al., 2018).

While it is understood that neurobiological changes in the hippocampus are a common consequence of aging, there is evidence that the rate of decline and overall volume loss is on a continuum from no cognitive impairment to MCI to AD and that differences are quantitative rather than qualitative (e.g., Jack et al., 2000; Fjell et al., 2013; Bangen et al., 2018).

The objective of this systematic review is to summarize the published literature relating to hippocampal volume and hippocampal atrophy in people without cognitive impairment, with MCI, and with AD. Specifically, this systematic review asked the question: Are the hippocampal changes observed prior to the onset of cognitive impairment parallel to those found in MCI and AD?

## Methods

This systematic review was guided by the Preferred Reporting Items for Systematic Reviews and Meta-Analyses (PRISMA) methodology (Page et al., 2021). Although the protocol was not prospectively registered, the literature search was conducted based on predetermined search terms and inclusion and exclusion criteria.

### Search strategy and data source

Literature searches were run in PubMed® up to September 15, 2022. The search terms used were: “atrophy” AND “hippocamp*” AND “Alzheimer’s disease” AND “Aging” AND “Magnetic Resonance Imaging” and prefiltered for human studies only. The asterisk was used as a wildcard symbol that broadened the search by identifying all words that start with “hippocamp” (e.g., hippocampus, hippocampal, hippocampi).

The titles of all returned publications were initially screened for non-English language and duplicate records. Next, two independent reviewers screened the titles and abstracts to identify studies for full text review. The full text and Supplementary materials of potential studies were obtained and screened for inclusion by two teams of two independent reviewers.

### Inclusion and exclusion criteria

The eligible study populations, interventions, comparators, outcomes, and study designs (PICOS) for the literature review were publications that: (a) were peer-reviewed, primary literature; (b) evaluated the adult human hippocampus or hippocampal subfields using 1.5 T magnetic resonance imaging (MRI) or greater for volume or 3 T MRI for structure; (c) assessed brain changes longitudinally for 1 year or longer; (d) included both healthy, cognitively normal, aged subjects and subjects with a diagnosis of AD and/or MCI; and (e) included subjects aged (on average) 65 years or older at baseline/first scan.

Studies were excluded if they failed to meet any of the inclusion criteria described above, as well as those that: (a) evaluated the hippocampus in dementias other than AD/MCI (e.g., Lewy body dementia, vascular dementia, frontotemporal dementia, tauopathy, Parkinson’s dementia) or Down’s syndrome with dementia; (b) included a mixed sample of different dementias; (c) evaluated changes in the hippocampus due to toxin exposure, head injury, ischemia, or any disease other than AD/MCI; or (d) assessed the effects of interventions or therapeutics. Narrative reviews, systematic reviews, meta-analyses, epidemiological studies, protocols, case studies, editorials and commentaries, retracted articles, theory or hypothesis papers, modeling or artificial intelligence papers, method/technology development or validation papers, conference abstracts/proceedings, doctoral dissertations, master’s theses, book chapters, and retracted publications were not included.

### Study selection

**Figure 1:**
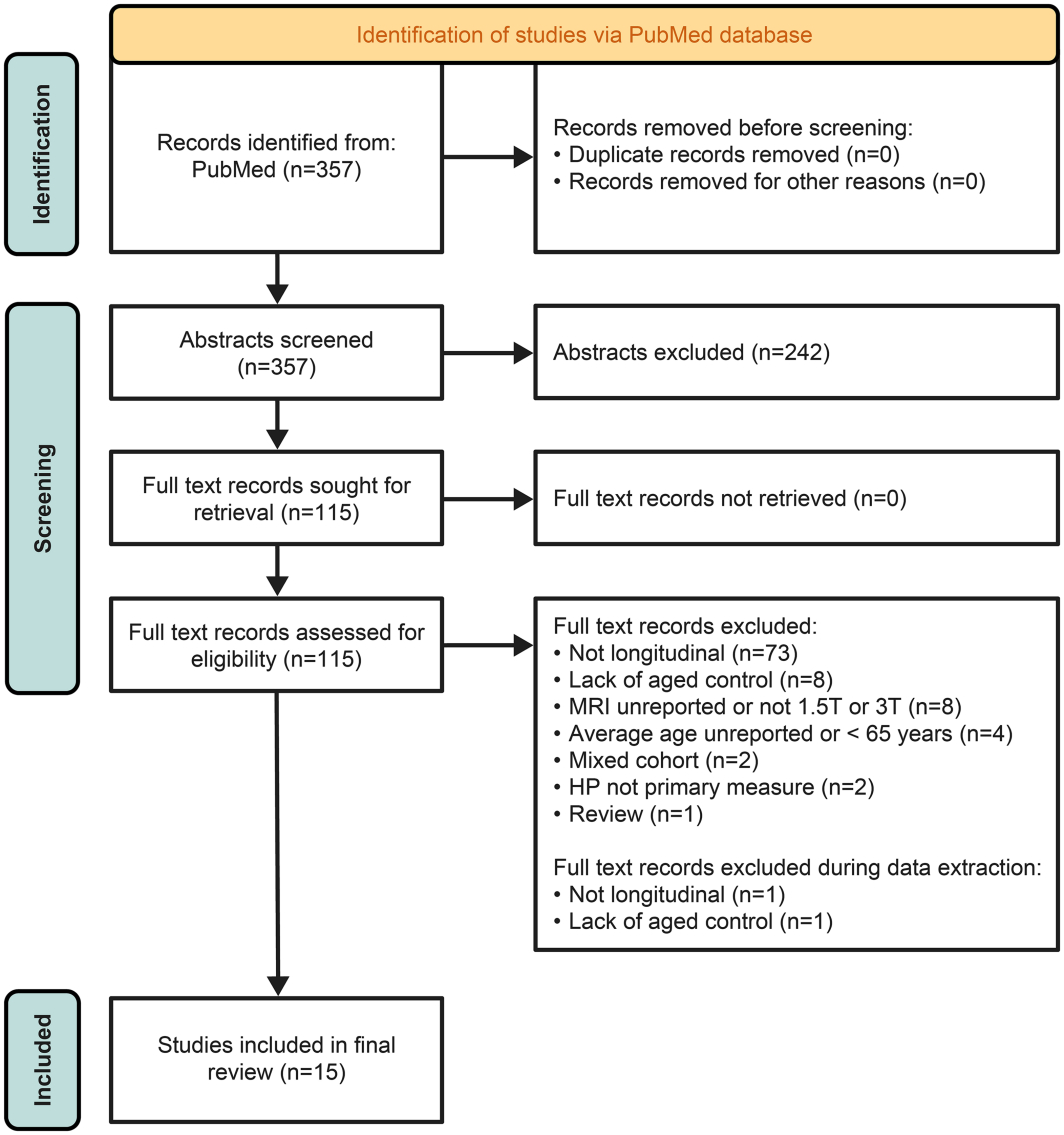
Search results.

All retrieved studies were assessed against the eligibility criteria. Primary screening of titles and abstracts was performed by two independent reviewers who reviewed each reference identified by the literature search to identify studies eligible for full text review. Any uncertainty regarding inclusion/exclusion decisions was resolved by consensus. For secondary screening of potentially relevant articles, full text articles and Supplementary material were obtained. Screening was performed by two teams of two independent reviewers. Any uncertainty regarding inclusion/exclusion decisions was resolved by consensus. The results of the study selection process are summarized in a PRISMA flowchart (Figure 1).

### Data extraction and synthesis

A standardized evidence data extraction template was developed in Microsoft Excel and populated with data extracted from the included studies. For each study, data were extracted from both the main study and Supplementary materials (when appropriate). All data were extracted, and quality checked against the original source article by a single reviewer. This systematic review synthesizes and summarizes the findings as they relate to hippocampal changes in people without cognitive impairment compared with those observed in MCI and in AD.

### Risk of bias assessment

The risk of bias assessment and methodological quality assessment was conducted using the US National Heart, Lung, and Blood Institute 14-item checklist for observational cohort and cross-sectional studies that assesses study population, risk, data presentation and analysis, study design, and outcome (National Institutes of Health, 2021). The quality assessment was performed by a single reviewer, with a quality check conducted by a second reviewer. The overall quality of eligible studies was determined using a scoring method previously described by Cotelli et al. (2020), in which the number of affirmative responses to 14 quality assessment questions (Supplementary Table S1) received one point and the sum of all responses was classified as follows: scores from 1 to 4 were rated as “poor,” 5–9 as “fair,” and 10–14 as “good.” A higher rating translated into a lower risk of bias.

## Results

### Study selection

The systematic search of the PubMed database yielded 357 articles (Figure 1). In total, 115 studies passed the primary title and abstract screening criteria and were retrieved and reviewed in full (Supplementary Tables S2, S3). Of these, 98 articles were excluded, with the most common reason for exclusion being MRI not longitudinal >1 year (*n* = 73; Figure 1). During data extraction, two additional studies were found to be unsuitable for the final systematic review [MRI not longitudinal >1 year (*n* = 1); lack of an aged control group (*n* = 1)]. The remaining 15 studies were included in the final systematic review (Figure 1).

### Study summary

The 15 included studies are detailed in Table 1. Based on the inclusion criteria, all studies had longitudinal MRI obtained from cognitively unimpaired subjects and those with a diagnosis of AD and/or MCI. It is important to note that 12 of the 15 studies analyzed existing data that were obtained from the databases of the Alzheimer’s Disease Neuroimaging Initiative (ADNI, *n* = 9), the Knight Alzheimer’s Disease Research Center (*n* = 1), the Oregon Aging and Alzheimer’s Disease Center at Oregon Health and Science University (*n* = 1), or the Mayo Clinic Alzheimer’s Disease Research Center and Alzheimer’s Disease Patient Registry (*n* = 1), and the other three studies were smaller observational studies.

**Table 1:** Studies included in the systematic review.

Study	Scan interval	MRI	N/Group	Average age (% Male)	Key outcomes related to HP change	RoB rating	Study limitations
Andrawis et al. (2012)	Baseline and 1-year follow-up	1.5 T	*N* total = 484 NC, *n* = 145 MCI, *n* = 243 AD, *n* = 96	Parental history (pooled cohort): MH+/PH- = 74.1 (52.3% M) PH+/MH- = 75.2 (54% M) MH+/PH+ = 76.3 (61.1% M) MH-/PH- = 74.8 (62.2% M)	Although NC subjects with MH+/PH- had smaller HP volumes at baseline (mean absolute difference 7.2% left, 7.4% right) and follow-up (mean absolute difference 8.4% left, 5.3% right) compared to subjects with MH-/PH+, differences were not statistically significant. MCI subjects with MH+/PH any had smaller right HP volumes at baseline (mean absolute difference 6%) and follow-up (mean absolute difference 9.5%) and greater atrophy on the right. AD subjects with MH+/PH any had larger left HP volumes at baseline (mean absolute difference 9%) and follow-up (mean absolute difference 14%) In the pooled cohort, MH+/PH any subjects had smaller right HP volumes at baseline and follow-up, relative to MH-/PH any subjects.	Good (10/14)	Parental history of dementia was self-reported.
Fiford et al. (2018)	Baseline up to 36 months, at 6-month intervals	1.5 T	*N* total = 683 NC, *n* = 191 MCI, *n* = 339 AD, *n* = 153	NC = 75.9 (51.8% M) MCI = 75.0 (62.5% M) AD = 75.0; (54.2% M)	AD subjects had average atrophy rates of 14 mL/year for the whole brain and 0.2 mL/year for HP. MCI (APOE negative) subjects had average rates of 10 mL/year for the whole brain and 0.1 mL/year for the HP. NC subjects (APOE negative) had average atrophy rates of 6 mL/year for the whole brain and 0.06 mL/year for the HP, and greater age at baseline was associated with significantly increased HP atrophy rate (0.03 mL/year for a 10-year increase). In NC, higher HP atrophy rates with age were observed and a reduction in HP atrophy rate with age was observed for AD.	Good (11/14)	Length of follow-up was significantly different in AD subjects. A small number of AD patients aged less than 65 years were included in the study. All patients had memory problems associated with a late-onset phenotype.
Insel et al. (2015)	Baseline up to 4 years, at 6-month intervals	1.5 T 3 T	*N* total = 291 NC, *n* = 108 MCI, *n* = 183	*N* total = 74.8 (61% M) NC = 75.5 (52% M) MCI = 74.4 (66% M)	In MCI, periods of accelerated atrophy were evident in several brain regions (including HP) over the course of CSF Aβ accumulation. Raw values not provided. Although a high number of control subjects were beyond the clinical threshold for Aβ positivity, excess Aβ accumulation did not necessarily result in an immediate increase in atrophy rate.	Fair (9/14)	Not all subjects had multiple MRI scans. Various follow-up rates. Limited Aβ42 data.
Jack et al. (2005)	Baseline and 1–2-year follow-up	1.5 T	*N* total = 163 NC, *n* = 91 aMCI, *n* = 72	At 2nd scan: NC = 81.9 (39.6% M) aMCI = 80.0 (56.9% M)	Rates of atrophy for MCI subjects were greater than NC subjects, including HP (−3.3 vs. -1.7 average annual % change). During follow-up, 39 subjects with MCI progressed to AD while 13 NC subjects developed MCI (11) or AD (2). For NC, only larger ventricular annual percent volume change was associated with a higher risk of conversion.	Good (11/14)	Some subjects had less than 1-year follow-up (NC, 1.4 years [0.9 to 2.0], MCI, 1.3 years [0.7 to 2.6]).
Li et al. (2011)	Baseline and at least 1-year follow-up, up to 7 years	1.5 T	*N* total = 150 NC, *n* = 72 AD, *n* = 64, plus 14 NC subjects who converted to dementia	NC = 71.8 (26.4% M) AD = 77.0 (51.3% M)	(A) Initial gray matter deficits were generally bilateral, located in anterior regions of the HP and entorhinal cortex. By time point two, there was a progression of atrophy in the medial temporal regions. HP gray matter loss extended so that most of the structure was affected. (B) There was variation across the AD subjects in terms of yearly rate of gray matter atrophy for both HP and MTG, but it was typically 1% for HP and 0.5% for MTG, which was, respectively, 2x and 5x greater than atrophy rate in NC. Atrophy rate was significantly higher in the MTG for the converter group. (C) For both the HP and MTG, local changes in gray matter concentration were the best predictors of future changes.	Fair (9/14)	The scan interval between the first and second time points was not the same for all the subjects. Combined data from AD and converter subjects.
Liu et al. (2021)	Baseline, with average of 2.1 years to last scan	1.5 T 3 T	*N* total = 1,523 NC, *n* = 421 Stable-MCI, *n* = 557 Converted to AD-MCI, *n* = 241 AD, *n* = 304	*N* total = 73.8 (54.2% M) NC = 74.0 (47.3% M) Stable-MCI = 73.0 (57.6% M) Converted to AD-MCI = 73.8 (58.9% M) AD = 75.0 (53.6% M)	Almost all HP subfields showed an increase in atrophy rate (%/year): NC (≤ 1%) < stable-MCI < converted-MCI < AD. Raw values not provided. Atrophy rate of whole HP was greater in stable-MCI compared to NC subjects and converted-MCI compared to stable-MCI and comparable between converted-MCI and AD. The presubiculum, dentate gyrus, and fimbria showed greater atrophy beyond the whole HP in HC, stable-MCI, and AD groups, and corresponded to a greater decline of memory and attention in the stable-MCI group.	Good (10/14)	Subjects had 1–12 scans. Various follow-up rates. Mix of 1.5 T (ADNI-1) MRI and 3 T (ADNI-2, ADNI-GO) MRI.
Lo et al. (2011)	Baseline up to 36 months, at 6- or 12-month intervals	1.5 T	*N* total = 819 NC, *n* = 229 MCI, *n* = 397 AD, *n* = 193	NC = 75.1 (52% M) MCI = 74.0 (64.5% M) AD = 74.6 (52.8% M)	The rate of HP atrophy was significantly slower in NC subjects with normal cognition (−2.95 mm^3^/month) than MCI (−5.52 mm^3^/month) or AD (−8.01 mm^3^/month), and slower in MCI than AD.	Good (11/14)	NC or MCI were followed-up over 3 years, whereas AD subjects were followed-up over 2 years maximum.
					APOE4 status was associated with Aβ42 in CSF (but not baseline HP volume) in NC. The presence of APOE4 accelerated HP atrophy in MCI and AD subjects.		There was a varying number of repeated measures for longitudinal analyses across different biomarkers and diagnostic groups.
Mattsson et al. (2014)	Baseline up to 4 years, ≤ 6 time points	1.5 T	*N* total = 62 NC Aβ-stable, *n* = 13 NC Aβ- declining, *n* = 13 NC Aβ+, *n* = 21 AD Aβ+, *n* = 15	NC Aβ-stable = 76.2 (76.9% M) NC Aβ-declining = 78.1 (38.5% M) NC Aβ + = 76.0 (57.1% M) AD Aβ + = 73.0 (40% M)	Compared with Aβ-stable subjects, Aβ-declining subjects had increased atrophy rates in overall, frontal, and parietal regions, and Aβ + subjects had increased atrophy rates in overall, amygdala, temporal, cingulate, frontal, and parietal regions. Raw values not provided. AD Aβ+ subjects had increased atrophy rates in all regions compared with Aβ- stable subjects and had increased atrophy rates in overall, HP, temporal, and cingulate regions compared with Aβ + subjects.	Fair (8/14)	Small sample size. Not all participants had MRI data (baseline [2], 6 months [4], 12 months [4], 24 months [11], 36 months [33], 48 months [54]) or CSF data.
Nosheny et al. (2015)	Baseline up to 4 years	1.5 T	*N* total = 567 NC, *n* = 208 MCI, *n* = 359	NC = 75.79 (51% M) MCI = 74.61 (63.2% M)	(1) In NC subjects, Aβ levels were significantly correlated with HP atrophy rate (mm^3^/year) when adjusting for age, gender, and APOE ε4 genotype. Raw values not provided. (2) In MCI, Aβ levels and APOE ε4+ genotype were associated with HP atrophy rate. (3) There was a significant HP atrophy rate in both Aβ+ and Aβ- NC subjects and MCI, and Aβ+ had a significantly higher HP atrophy rate than Aβ-. (4) In MCI or the combined cohort, there was a significant interaction between age and Aβ status, with a stronger association between age and HP atrophy rate in the Aβ- subjects. (5) Less than half of the age-related HP atrophy rate was attributable to Aβ levels in NC subjects, MCI, or the combined cohort.	Fair (9/14)	A significantly greater number of male than female subjects with MCI compared to normal controls. CSF amyloid-β samples were taken before the initial volume measurements.
Silbert et al. (2003)	Followed for ~5.8 years to autopsy; ~28 months from last MRI to death	1.5 T	*N* total = 39 NC, *n* = 15 Cognitive impairment, *n* = 24	*N* total = 84.00 (54% M) NC = 87.90 (53.3% M) Cognitive impairment = 81.53 (54.2% M)	In subjects with cognitive impairment, there was a relationship between the degree of HP neurofibrillary tangle burden and total HP volume prior to death. No such relationship existed between the amount of neurofibrillary tangle and the rate of HP volume change.	Good (10/14)	Small sample size. Not all subjects had multiple MRI scans.
					In NC subjects, no significant relationship was found between HP neurofibrillary tangle burden and last HP volume prior to death or rate of HP volume change over time. In cognitively impaired subjects, the average rate of ventricular volume increase was 5.5 cc/year, while the average rate of CNS volume decrease was −17.9 cc/year. NC subjects showed an average increase of 3.3 cc/year in ventricular volume size and −1.8 cc/year in total brain volume over time.		Various follow-up rates. Two of the 39 subjects had mixed AD and vascular pathology.
Skup et al. (2011)	Baseline up to 3 years	1.5 T	*N* total = 687 NC, *n* = 224 aMCI, *n* = 266 Probable AD, *n* = 197	NC = 76.00 (50.9% M) aMCI = 74.91 (66.2% M) Probable AD = 75.65 (51.3% M)	Validation findings showed that AD and aMCI subjects had decreased HP volume over time compared to NC. Raw values not provided. Male and female AD and aMCI subjects showed different patterns of decline over time compared to control subjects in bilateral precuneus, bilateral caudate nucleus, right entorhinal gyrus, bilateral thalamus, bilateral middle temporal gyrus, left insula, and right amygdala.	Good (11/14)	NC or MCI were followed-up over 3 years, whereas AD subjects were followed-up over 2 years. Male patients were over-represented among the aMCI cohort. Data was utilized from stable AD subjects with early cognitive decline and stable aMCI subjects who were not separated into subtypes.
Tarawneh et al. (2015)	Baseline and 2- to 3-year follow-up	1.5 T 3 T	*N* total = 87 NC, *n* = 64 AD, *n* = 23	*N* total = 72.6 (at lumbar puncture) NC = 72.3 (29.7% M) AD = 73.6 (39.1% M)	At baseline, average HP volume for NC (7,438 mm^3^) significantly differed from AD (6,310 mm^3^). In AD, baseline levels of VILIP-1, tau, and p-tau predicted whole-brain and HP atrophy. Cognitively NC subjects with CSF markers in the upper tercile had higher rates of whole-brain and HP atrophy compared to those with lower levels (adjusting for age, sex, imaging system type, and APOE ε4 genotype). CSF biomarker levels and rates of whole-brain and regional atrophy in this subset of controls were similar to AD subjects.	Fair (8/14)	Small sample size. Short and varied follow-up. Mix of 3 T and 1.5 T for structural MRI.
					The mean (SE) adjusted rate of atrophy in the control cohort was −0.003 (0.001) points per year for normalized whole-brain volume (−0.4% annual change from baseline), −100 (25) mm^3^ per year for HP volume (−1.3% annual change from baseline), and −0.05 (0.02) mm per year for entorhinal thickness (−1.3% annual change from baseline).		
Wang et al. (2009)	Baseline and up to 2-years, 12-month intervals	1.5 T	*N* total = 78 NC, *n* = 20 Stable aMCI, *n* = 39 Progressive aMCI, *n* = 19	NC = 75.1 (55% M) Stable aMCI = 75.6 (79.5% M) Progressive aMCI = 77.6 (63.2% M)	Hippocampal volumes differed significantly, with NC subjects > stable aMCI > progressive aMCI. Raw values not provided. Compared with NC subjects, progressive aMCI subjects had significantly greater atrophy rates in both right and left HP. Compared to stable aMCI subjects, progressive aMCI subjects had faster atrophy rate in right, but not left, HP. There were no APOE4 group effects on neuropsychological or MRI volumetric assessments. In MCI subjects, there was a significant correlation between the decline in memory scores and HP atrophy rates.	Good (10/14)	Small sample size. Varied follow-up period. Inclusion of more male than female subjects.
Whitwell et al. (2007)	Median interval between first MRI and AD conversion 3 years	1.5 T	*N* total = 66 NC, *n* = 33 aMCI converters to AD, *n* = 33	NC, median = 78 (42.4% M) aMCI converters to AD, median = 78 (42.4% M)	The pattern of gray matter loss in aMCI three years before conversion was primarily in the medial temporal lobes, including HP. The extent and magnitude of the cerebral atrophy further progressed by the time subjects were one year before conversion and extended to include posterior temporal lobe and the entire extent of HP. By the time subjects had a clinical diagnosis of AD the pattern of gray matter atrophy had become more widespread. Raw values not provided. The HP showed progressive atrophy throughout the disease course, with severity of HP loss detected on MRI increasing at each time point.	Good (10/14)	Small sample size. Variable scan interval.

Aβ, amyloid-β (+, −); AD, Alzheimer’s disease; ADNI, Alzheimer’s Disease Neuro-Imaging Initiative; APOE, apolipoprotein E; aMCI, amnestic mild cognitive impairment; CNS, central nervous system; CSF, cerebral spinal fluid; HP, hippocampus; M, male; MCI, mild cognitive impairment; MH, maternal history of dementia (+, −, any); MRI, magnetic resonance imaging; MTG, medial temporal gyrus; NC, normal control; OASIS, Open Access Structural Imaging Series; PH, paternal history of dementia (+, −, any); RoB, Risk of Bias; SE, standard error; VILIP-1, visinin-like protein 1.

The total sample sizes of the included studies ranged from 39 to 1,523 subjects. Overall, mean age of subjects was over 70 years of age. In general, scan intervals and follow-up were variable within samples and between studies; however, most studies had a follow-up period of two or more years and at least two scans per subject. The studies used 1.5 T MRI (n = 12), 3 T MRI (n = 1), or both (n = 2), and for the purpose of this systematic review, we focused on total hippocampus or hippocampal subfield volume and/or atrophy data. In all the included studies, cognitively normal-aged subjects were considered the control group.

Overall, there was large heterogeneity among these studies due to differences in study designs, measurements and methodologies to determine hippocampal changes, sample sizes, scan interval, and follow-up period, making direct comparisons difficult. For this reason, the results are presented below as summaries of each study.

### Results of individual studies

#### Alzheimer’s disease neuroimaging initiative studies

The following studies evaluated data from the ADNI database. ADNI enrolls participants between the ages of 55 and 90 years recruited from sites located in the United States and Canada. Participants undergo a series of initial tests that are repeated at intervals over subsequent years, including a clinical evaluation, neuropsychological tests, genetic testing, lumbar puncture, and MRI and positron emission tomography (PET) scans. Subjects are classified as normal subjects, MCI subjects and mild AD subjects. Normal subjects are defined as those with Mini-Mental State Examination (MMSE) scores between 24 and 30 (inclusive), a Clinical Dementia Rating (CDR) of 0, non-depressed, non-MCI, and nondemented. MCI subjects are defined as those with MMSE scores between 24 and 30 (inclusive), a memory complaint, have objective memory loss measured by education adjusted scores on Wechsler Memory Scale Logical Memory II, a CDR of 0.5, absence of significant levels of impairment in other cognitive domains, essentially preserved activities of daily living, and an absence of dementia. Mild AD subjects are defined as those with MMSE scores between 20 and 26 (inclusive), CDR of 0.5 or 1.0, and meet the National Institute of Neurological and Communicative Disorders and Stroke and the Alzheimer’s Disease and Related Disorders Association criteria for probable AD (NINCDS-ADRDA) (Alzheimer’s Disease Neuroimaging Protocol, 2016), which incorporates the use of biomarkers to determine different disease stages (Jack Jr et al., 2011).

Andrawis et al. (2012) examined the effects of maternal history of dementia in 243 subjects with MCI, 96 subjects with AD, and 145 normal-aged control subjects from the ADNI database (Table 1). All subjects were scanned using 1.5 T MRI at baseline and 1-year follow-up. Overall, MCI subjects with positive maternal history of dementia had smaller hippocampal volumes at baseline and follow-up, and greater 12-month atrophy rates than subjects with negative maternal history. Although those without cognitive impairment and a positive maternal history/negative paternal history of dementia had smaller hippocampal volumes at baseline and follow-up compared to subjects with positive paternal history and maternal negative history of dementia, these differences were not statistically significant. A limitation of this study is the self-reported parental history of dementia.

Fiford et al. (2018) studied patterns of whole-brain and hippocampal atrophy in 339 subjects with MCI, 153 subjects with AD, and 191 without cognitive impairment from the ADNI database (Table 1). All subjects were scanned using 1.5 T MRI at baseline and at 6-month intervals, up to 36 months, atrophy rates were calculated based on longitudinal volume change. AD subjects had average atrophy rates of 14 mL/year for the whole brain and 0.2 mL/year for the hippocampus. Subjects with MCI had atrophy rates of 10 mL/year for the whole brain and 0.1 mL/year for the hippocampus, for apolipoprotein E (APOE) negative individuals. Subjects without cognitive impairment had average atrophy rates of 6 mL/year for the whole brain and 0.06 mL/year for the hippocampus, for APOE-negative individuals (APOE-positive individuals not reported). However, a significantly greater hippocampal atrophy rate was observed in older patients within the control group and in younger patients with AD. Limitations of this study include significantly different follow-up rates in AD vs. normal control subjects, and various methodological limitations, such as the fact that there was a small number of AD patients aged less than 65 years and all patients had memory problems associated with a late-onset phenotype.

Insel et al. (2015) estimated the association between cerebral spinal fluid (CSF) amyloid-β and regional brain atrophy in 183 subjects with MCI and 108 control subjects from the ADNI database (Table 1). All subjects were scanned using 1.5 T MRI at baseline and at 6-month intervals, up to 4 years. In MCI, periods of accelerated atrophy were evident in several brain regions, including the hippocampus, well before CSF levels of Aβ42 reached the threshold for “preclinical AD.” Additionally, although a substantial number of subjects with unimpaired cognition were above the threshold of amyloid-β positivity, excess accumulation of amyloid-β alone did not necessarily result in an immediate increase in atrophy rates and cognitive impairment. This could be due to latent factors such as the duration of exposure to amyloid-β pathology or concurrent co-pathologies such as Lewy body pathology and cardiovascular disease. These factors may contribute to an increased vulnerability of MCI subjects to the effects of amyloid-β, in contrast to controls. Overall, CSF levels of Aβ42 were associated with APOE4 positivity and Alzheimer’s Disease Assessment Scale-cognitive subscale (ADAS-cog) within both groups. Limitations of this study include various follow-up rates, limited Aβ42 data, and the fact that not all subjects had multiple MRI scans up to 4 years.

Jack et al. (2005) hypothesized that atrophy rate was associated with time to subsequent clinical diagnosis to a more impaired cognitive state in both cognitively unimpaired subjects (*n* = 91) and those with amnestic MCI (aMCI; *n* = 72) from the ADNI database (Table 1). All subjects were scanned using 1.5 T MRI at baseline and 1- and 2-year follow-up. Rates of atrophy for subjects with MCI were greater than for those with unimpaired cognition, including in the hippocampus (−3.3 vs. −1.7 average annual % change). During follow-up, 39 subjects with MCI progressed to AD while 13 with unimpaired cognition developed MCI (*n* = 11) or AD (*n* = 2). For the subjects with unimpaired cognition, only larger ventricular annual percent volume change was associated with a higher risk of conversion. Limitations of this study include relatively short and varied years of patient follow-up.

Liu et al. (2021) correlated hippocampal subfield atrophy with CSF biomarkers and cognitive decline over the course of AD in 557 subjects with stable MCI, 304 subjects with AD, 241 subjects who progressed from MCI to AD, and 421 subjects with unimpaired cognition from the ADNI database (Table 1). All subjects were scanned using 1.5 T or 3 T MRI at baseline and had, on average, 2.1 years between the first and last scan. Almost all hippocampal subfields showed an increase in percent atrophy rate per year (control < stable MCI < developed MCI < AD); the same pattern of atrophy severity was observed in the whole hippocampus. Compared with the whole hippocampus, the presubiculum and molecular layer of the dentate gyrus showed a greater atrophy rate in all four groups. In the subjects with stable MCI and those who progressed from MCI to AD, a decline in attention was associated with the atrophy rate of most of the subicular complex and CA regions. In incident MCI, presubiculum atrophy was associated with CSF tau levels and corresponded to the onset age of AD and a decline in attention. Limitations of this study include use of a mix of 1.5 T and 3 T MRI, various follow-up rates, and the fact that not all subjects had multiple MRI scans.

Lo et al. (2011) assessed the relationship between the longitudinal change of biomarkers (e.g., Aβ42 level in CSF) and hippocampal volume, as well as the presence of the APOE4 gene, on cognitive decline in 193 subjects with AD, 397 subjects with MCI, and 229 normal-aged control subjects from the ADNI database (Table 1). All subjects were scanned using 1.5 T MRI at baseline and 6- or 12-month intervals, up to 36 months. The rate of hippocampal atrophy was significantly slower in control subjects with normal cognition (−2.95 mm^3^/month) than in those with MCI (−5.52 mm^3^/month) or AD (−8.01 mm^3^/month). The Aβ42 level appeared to decrease faster in control subjects than in those with MCI or AD, although the differences did not reach statistical significance. In normal control subjects, APOE4 status was associated with Aβ42 in CSF but not baseline hippocampal volume, and in subjects with MCI and AD, APOE4 was associated with accelerated hippocampal atrophy. For subjects with MCI, changes on the ADAS-cog were associated with a decrease in the Aβ42 level in the CSF and hippocampal volume. Limitations of this study include longer follow-up rates for normal control or MCI subjects than for those with AD and the varying number of repeated measures for longitudinal analyses across different biomarkers and diagnostic groups.

Mattsson et al. (2014) sought to determine whether the development of amyloid-β pathology is related to increased regional atrophy in the brains of 47 control subjects (*n* = 13, amyloid-β- stable; *n* = 13, amyloid-β- declining; *n* = 21, amyloid-β+) and 15 subjects with AD from the ADNI database (Table 1). All subjects underwent repeated CSF Aβ42 measurements and 1.5 T MRI at baseline and at 6-, 12-, 24-, 36-, and 48-months. At baseline, amyloid-β- stable control subjects had smaller overall, hippocampus, amygdala, temporal, control, and parietal volumes compared with amyloid-β+ normal subjects. In addition, compared with amyloid-β- stable normal subjects, amyloid-β- declining control subjects had increased atrophy rates in overall, frontal, and parietal regions, and amyloid-β+ control subjects had increased atrophy rates in overall, amygdala, temporal, cingulate, frontal, and parietal regions. The AD amyloid-β+ subjects had increased atrophy rates in all regions compared with control amyloid-β- stable normal subjects and had increased atrophy rates in overall, hippocampus, temporal, and cingulate regions compared with amyloid-β+ normal subjects, suggesting that atrophy may accelerate in the hippocampus, temporal, and cingulate regions as subjects progress to dementia. Limitations of this study include a small sample size and the fact that not all subjects had CSF and/or MRI at all timepoints.

Nosheny et al. (2015) evaluated factors that contributed to hippocampal atrophy rate in 359 MCI subjects and 208 clinically normal-aged subjects from the ADNI database (Table 1). All subjects were scanned using 1.5 T MRI over 4 years. In control subjects, only increased amyloid-β levels in the CSF were significantly associated with hippocampal atrophy rate when adjusting for age, gender, and APOE4 genotype. In MCI subjects, both amyloid-β levels and APOE4+ genotype were significantly associated with hippocampal atrophy rate. Rates of hippocampal atrophy were significant in amyloid-β+ and amyloid-β- normal subgroups for both control and MCI, with amyloid-β+ subjects having a higher hippocampal atrophy rate. In the MCI only or the combined cohort (controls+MCI), there was a significant interaction between amyloid-β status and age, with a stronger association between age and hippocampal atrophy rate in the amyloid-β- subjects indicating that age had a significantly higher association with hippocampal atrophy in amyloid-β- participants than in amyloid-β+ participants in MCI. Although the presence of amyloid-β was a major predictor of hippocampal atrophy rate, in most cases, the hippocampal atrophy rate was not associated with the presence of amyloid-β and thought to be a consequence of aging and other unknown factors. Limitations of this study include a significantly greater number of male than female subjects with MCI compared to normal controls, and the fact that CSF amyloid-β samples were taken before the initial volume measurements.

Skup et al. (2011) studied whether there were gender differences in gray matter atrophy patterns over time in 197 subjects with AD, 266 subjects with aMCI (defined as MCI with memory loss as the predominant symptom), and 224 control subjects from the ADNI database (Table 1). All subjects were scanned using 1.5 T MRI over 2 (AD group) and 3 years (aMCI and control group). Validation studies showed that AD and subjects with aMCI had decreased hippocampal volume over time compared with normal control subjects. Male and female subjects with AD and aMCI showed different patterns of decline over time compared with normal control subjects in bilateral precuneus, bilateral caudate nucleus, right entorhinal gyrus, bilateral thalamus, bilateral middle temporal gyrus, left insula, and right amygdala. Limitations of this study include longer follow-up for subjects with normal control or MCI than those with AD and various methodological limitations such as the over-representation of males among aMCI patients and the utilization of data from stable AD subjects who were relatively early in their cognitive decline as well as from individuals with stable aMCI who were not separated into subtypes.

#### Other studies

Li et al. (2011) investigated the gray matter changes in AD progression in 64 subjects with AD and 86 subjects with unimpaired cognition, including 14 who developed dementia during the course of the study, from the Open Access Series of Imaging Studies (OASIS) dataset generated across several projects through the Washington University School of Medicine Knight Alzheimer Disease Research Center (Table 1). The AD subjects were clinically diagnosed with very mild to moderate AD (CDR = 0.5, very mild dementia; CDR = 1, mild dementia; or CDR = 2, moderate dementia). All subjects were scanned using 1.5 T MRI at baseline and at least 1-year follow-up. Initial gray matter deficits encompassed the hippocampus and entorhinal cortex regions, with development of increasing pathology in other regions over time. Results suggested faster atrophy rates in the hippocampus over several years near illness onset. In general, there was variation across subjects with AD in terms of yearly rate of gray matter atrophy for both the hippocampus and medial temporal gyrus, but it was typically 1% for the hippocampus and 0.5% for medial temporal gyrus, which was, respectively, 2x and 5x greater than the atrophy rate in normal control subjects. However, the atrophy rate was significantly higher in the medial temporal gyrus for the converter group. Limitations of this study include differing time intervals between the first and second time points for all subjects, and the utilization of combined data from AD subjects and subjects who developed dementia during the course of the study.

Silbert et al. (2003) investigated whether changes in antemortem brain volume are predictive of subsequent AD pathology in 24 cognitively impaired subjects and 15 normal-aged control subjects from the Oregon Aging and Alzheimer’s Disease Center at Oregon Health and Science University (Table 1). Subjects were categorized according to their clinical dementia stage based on the last CDR as nondemented (CDR = 0.0) or demented (CDR = 0.5). Neuropathologic diagnosis of AD was determined using regional postmortem measures of neurofibrillary tangles. All subjects were followed on average for 5.8 years and scanned using 1.5 T MRI, with an average interscan interval of 4.1 years. In subjects with cognitive impairment, there was a relationship between degree of hippocampal neurofibrillary tangle burden and total hippocampal volume prior to death. However, no such relationship was observed between the amount of neurofibrillary tangle burden and the rate of hippocampal volume change. In normal control subjects, no significant relationship was found between hippocampal neurofibrillary tangle burden and hippocampal volume prior to death or rate of hippocampal volume change over time. In cognitively impaired subjects, the average rate of ventricular volume increase was 5.5 cc/year, while the average rate of central nervous system volume decrease was −17.9 cc/year. In normal control subjects, the average rate of ventricular volume increase was 3.3 cc/year, while the average rate of total brain volume decrease was −1.8 cc/year. Limitations of this study include various follow-up rates, a small sample size, the inclusion of two subjects with mixed AD/vascular pathology, and the fact that not all subjects had multiple MRI scans.

Tarawneh et al. (2015) studied the usefulness of various CSF markers (e.g., Aβ42, p-tau) in predicting rates of whole-brain neurodegeneration and whole-brain atrophy in 23 subjects with mild AD and 64 cognitively normal-aged control subjects from The Charles F. and Joanne Knight Alzheimer’s Disease Research Center at Washington University School of Medicine (Table 1). All individuals in the AD cohort had a clinical diagnosis of very mild symptomatic AD (CDR = 0.5) at the baseline assessment. APOE genotypes were obtained. All subjects had baseline CSF biomarker measurements and were scanned using 1.5 T or 3 T MRI at baseline and 2- to 3-year follow-up. At baseline, average hippocampal volume for normal control subjects (7,438 mm^3^) significantly differed from subjects with AD (6,310 mm^3^). The average adjusted rate of atrophy in normal control subjects was −0.003 points per year for normalized whole-brain volume (−0.4% annual change from baseline) and −100 mm^3^ per year for hippocampal volume (−1.3% annual change from baseline), whereas the adjusted rate of atrophy in AD was −0.007 points per year for normalized whole-brain volume (−0.9% annual change from baseline) and −271 mm^3^ per year for hippocampal volume (−4.3% annual change from baseline). In subjects with AD, baseline levels of certain CSF markers (VILIP-1, tau, p-tau) predicted whole-brain and hippocampal atrophy. After adjusting for age, sex, imaging system type, and APOE 4 genotype, cognitively normal control subjects with CSF markers in the upper tercile had higher rates of whole-brain and hippocampal atrophy compared to those with lower levels of CSF markers. In this subset of normal control subjects, CSF biomarker levels and rates of whole-brain and regional atrophy were similar to subjects with AD. Limitations of this study include a relatively short and varied follow-up, a small sample size, and the use of a mix of 1.5 T and 3 T for structural MRI.

Wang et al. (2009) researched hippocampal atrophy rates in 39 subjects with stable aMCI, 19 subjects with progressive aMCI, and 20 control subjects from Taipei Veterans General Hospital (Table 1). Clinical diagnoses were based on medical history, plus clinical and neuropsychological assessment. Subjects diagnosed with MCI fulfilled Petersen’s criteria of aMCI, meaning they were nondemented, had subjective memory complaints, objective memory impairment, normal general cognitive function, intact daily living activities, and a CDR score of 0.5. All subjects were scanned using 1.5 T MRI at baseline and at 12-month intervals, up to 2 years. At baseline, bilateral hippocampal volumes differed significantly between the groups (normal control subjects > stable MCI > progressive MCI). During follow-up, subjects with progressive MCI had significantly greater atrophy rates in both the right and left hippocampus compared with normal controls, and greater atrophy in the right, but not left hippocampus, compared to subjects with stable MCI. In subjects with MCI, there was a significant correlation between decline in memory test scores and hippocampal atrophy rates. Limitations of this study include a small sample size, the inclusion of more male than female subjects, and a variable follow-up period.

Whitwell et al. (2007) assessed the progression of cerebral atrophy during conversion to AD in 33 subjects with aMCI converting to AD and 33 control subjects from the Mayo Clinic Alzheimer’s Disease Research Center and Alzheimer’s Disease Patient Registry (Table 1). Individuals were diagnosed as having aMCI if they met the following criteria: (1) memory complaint, preferably corroborated by an informant; (2) memory impairment for age; (3) essentially normal general cognitive function; (4) generally preserved activities of daily living; (5) not demented. All subjects were scanned using 1.5 T MRI three times, approximately 3 years before conversion from aMCI to AD, approximately 1 year before conversion, and at the time of conversion. 3 years prior to conversion from aMCI to AD, gray matter loss was primarily observed in the medial temporal lobe, including the bilateral anterior hippocampus. The magnitude of cerebral atrophy progressed by the time the subjects were 1 year prior to conversion and extended to include the posterior temporal lobe and entire hippocampus. By the time subjects had a clinical diagnosis of AD, the pattern of gray matter atrophy had become more widespread, with severe gray matter loss throughout the temporal lobes, in the temporoparietal association neocortex, and in the frontal lobes. Overall, the hippocampus showed progressive atrophy throughout the disease course, with the severity of volume loss detected on MRI increasing at each time-point measured. Limitations of this study include a small sample size and a variable follow-up period.

### Quality of the included studies

The risk of bias rating was “good” for nine of the 14 included studies, while a “fair” risk of bias rating was obtained for the remaining five studies (Supplementary Table S1). These results indicated a low overall risk of bias in the included evidence, owning to the well-defined methodologies and use of robust databases with longitudinal data. A common limitation observed in the evidence was the lack of sample size justification, which is inherent in observational studies due to their exploratory nature.

## Discussion

In this systematic review, we summarized the evidence related to hippocampal changes detected by longitudinal MRI in people with no cognitive impairment, those with MCI and those with AD populations from 15 studies published between 2003 and 2021, including nine studies from ADNI. Despite the large heterogeneity among these studies due to differences in study designs, methodologies, sample sizes, scan intervals, and follow-up period, this systematic review provides further support for the view that hippocampal changes, specifically decreased volume and increased rate of atrophy, occur during aging, MCI and AD, with greater changes in the latter two.

All studies included cognitively unimpaired, aged subjects and those with MCI and/or AD. Several of the studies observed that the hippocampus and hippocampal subfields change over time, with changes in AD greater than changes in MCI, and changes in MCI greater than changes prior to cognitive impairment (e.g., Jack et al., 2005; Wang et al., 2009; Lo et al., 2011; Skup et al., 2011; Fiford et al., 2018; Liu et al., 2021). In general, the hippocampus showed progressive atrophy near the onset of AD (Li et al., 2011) and throughout the disease course (Whitwell et al., 2007), and was associated with cognitive decline (Lo et al., 2011;). In subjects with dementia, a relationship was observed between the degree of hippocampal neurofibrillary tangle burden and total hippocampal volume prior to death (Silbert et al., 2003). Finally, maternal history of dementia had a significant effect on hippocampal atrophy in MCI and AD and was non-significantly associated with smaller hippocampal volumes in cognitively unimpaired controls (Andrawis et al., 2012).

Various hippocampus outcomes were reported for those without cognitive impairment. In particular, the percent yearly atrophy rate in aged unimpaired subjects was reported at approximately 1% (e.g., Jack et al., 2005; Li et al., 2011; Tarawneh et al., 2015; Liu et al., 2021) and older age at baseline was associated with hippocampal atrophy rate (Fiford et al., 2018). In multiple studies, amyloid-β status (Mattsson et al., 2014) or amyloid-β levels (Nosheny et al., 2015) were associated with hippocampal atrophy rate. However, it was also shown that in control subjects, excess accumulation of amyloid-β beyond the clinical threshold for amyloid-β positivity did not necessarily result in an immediate increase in atrophy rates and cognitive impairment (Insel et al., 2015). Notably, Nosheny et al., 2015 concluded that the presence of brain amyloid is a major predictor of hippocampal atrophy rate in older adults, but that in most cases, hippocampal atrophy rate in older adult subjects with unimpaired cognition or MCI is not associated with the presence of amyloid-β and is likely to be a consequence of aging and other unknown factors.

Finally, we are aware of at least one study related to hippocampal changes in cognitively unimpaired subjects published since the literature search for this systematic review was conducted and was therefore not included above (Huang et al., 2023). The results reported by Huang et al. (2023) provide further support that hippocampal atrophy observed in patients with AD and MCI is faster than atrophy during normal aging.

The findings from the studies included in this review support the view that the mechanisms of hippocampal atrophy resulting in synaptic damage and neuronal loss are likely shared between AD and healthy aging (Crews and Masliah, 2010). The proposed key stressors in this process are oxidative stress caused by free radicals and inflammation (Kao et al., 2020; Zia et al., 2021; Andronie-Cioara et al., 2023; Plascencia-Villa and Perry, 2023), both rapidly affecting the hippocampus, known for its high plasticity, and therefore its vulnerability toward stressors (McEwen, 1994).

The strength of this systematic review is that it was guided by the PRISMA methodology. However, a critical appraisal of the included studies to qualitatively assess the robustness of individual study methodologies and their reporting standards was not performed.

Furthermore, due to the restrictive nature of the search criteria, the search may have missed publications that looked at longitudinal hippocampal changes in cognitively unimpaired aging or individuals with (non-clinical) subjective cognitive decline as the primary endpoint (in the absence of an AD group). Specifically, in the studies that met the inclusion criteria, normal aging subjects were considered the control group. Thus, hippocampal changes in normal aging were not the focus of the studies.

It is also worth noting that nine of the publications used existing longitudinal data from the ADNI database across overlapping data collection timeframes. It is likely that the same patient pool might have been investigated across multiple publications. Furthermore, many of the publications did not report the specific timeframe of data collection. Therefore, the scope of data overlap across these studies is unclear, and an overrepresentation of evidence from these studies cannot be ruled out. Thus, we cannot consider these studies as being independent.

Finally, the publications used different imaging strengths, measurements, and methodologies to determine hippocampal changes, making direct comparisons of outcomes difficult and inappropriate.

Patterns of brain volume decline have been shown in patients treated with anti-amyloid therapies and therapeutic antibodies. Donanemab and lecanemab have been shown to increase ventricular volume and decrease brain volume, though hippocampal volume loss due to donanemab resembled that of placebo (Lowe et al., 2021; Swanson et al., 2021). In addition, the specific multinutrient intervention containing Fortasyn Connect™ (Souvenaid™, Nutricia, N.V., Zoetermeer, Netherlands) significantly slowed hippocampal volume and whole brain volume decline in a randomized controlled trial with 311 prodromal AD patients (Soininen et al., 2021). The results highlight the importance of hippocampal changes across the continuum from normal aging to AD.

## Conclusion

The findings of this systematic review suggest that hippocampal changes observed prior to the onset of cognitive impairment parallel those found in MCI and AD. In addition, the published literature included here indicates that the rate of hippocampal decline and extent of volume loss between those without cognitive impairment, MCI, and AD is quantitative rather than qualitative. The yearly hippocampal atrophy rate is approximately 1% in aging subjects with unimpaired cognition compared with approximately 3–5% in subjects with MCI and AD. This suggests that hippocampal atrophy, like cognitive function and AD pathology, is on a continuum and that the disease status, i.e., MCI and dementia, represents a cut point along a single disease process.

## Data Availability

The original contributions presented in the study are included in the article/Supplementary material, further inquiries can be directed to the corresponding author/s.
