# Supplementary material for: The relationship between hippocampal changes in healthy aging and Alzheimer’s disease: a systematic literature review
Source: Front Aging Neurosci. 2024 Aug 15;16:1390574. doi: 10.3389/fnagi.2024.1390574 (PMC11357962; doi:10.3389/fnagi.2024.1390574)
Supplement: Supplementary file 1 [file Table_1.docx]

Supplemental Material

The Relationship Between Hippocampal Changes in Healthy Aging and Alzheimer’s Disease: A Systematic Literature Review

**Michael Woodward*, MD^1^, David A. Bennett, MD^2^; Tatjana Rundek, MD PhD^3^;**
**George Perry, PhD^4^; Tomasz Rudka, PhD^5^**

***Correspondence:** Michael Woodward: [Michael.Woodward@austin.org.au](mailto:Michael.Woodward@austin.org.au)

# Supplemental Tables

## Supplemental Table S1. Study quality assessment


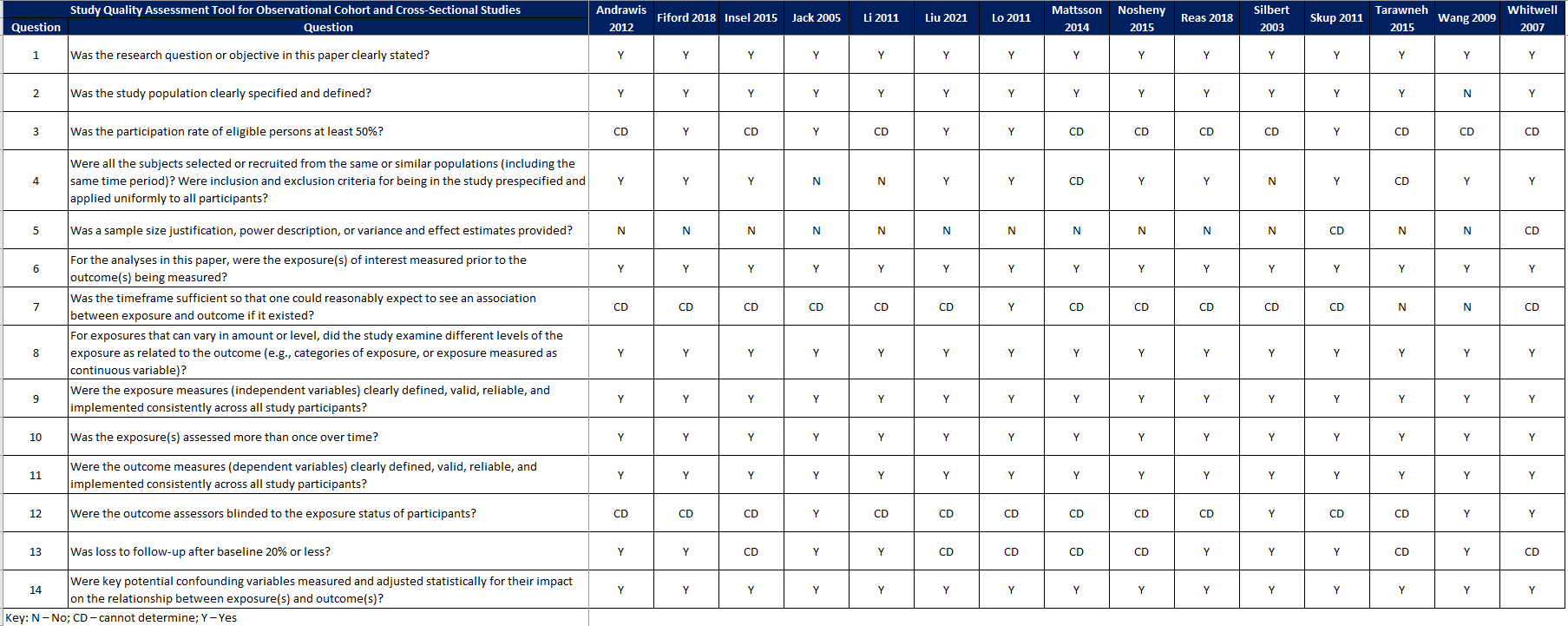


CD, cannot determine; N, no; Y, yes.

## Supplemental Table S2. Studies excluded during full text review based on inclusion and exclusion criteria

| **Study** | **Title** | **Primary Reason for Exclusion** |
| --- | --- | --- |
| Granger et al., 2022 | Hippocampal dentate gyrus integrity revealed with ultrahigh resolution diffusion imaging predicts memory performance in older adults | Not longitudinal > 1 year |
| Langella et al., 2021 | The association between hippocampal volume and memory in pathological aging is mediated by functional redundancy | Not longitudinal > 1 year |
| Qing et al., 2021 | Causal structural covariance network revealing atrophy progression in Alzheimer's disease continuum | Not longitudinal > 1 year |
| Chen et al., 2021 | Evaluating the association between brain atrophy, hypometabolism, and cognitive decline in Alzheimer's disease: a PET/MRI study | Average age unreported or < 65 years |
| Contrador et al., 2021 | Longitudinal brain atrophy and CSF biomarkers in early-onset Alzheimer's disease | Average age unreported or < 65 years |
| Belleville et al., 2020 | Neural correlates of resilience to the effects of hippocampal atrophy on memory | Not longitudinal > 1 year |
| Machado et al., 2020 | The cholinergic system in subtypes of Alzheimer's disease: an in vivo longitudinal MRI study | Mixed cohort |
| Koenig et al., 2020 | Select Atrophied Regions in Alzheimer disease (SARA): An improved volumetric model for identifying Alzheimer disease dementia | Not longitudinal > 1 year |
| Dawe et al., 2020 | Late-life cognitive decline is associated with hippocampal volume, above and beyond its associations with traditional neuropathologic indices | Average age unreported or < 65 years |
| Bettcher et al., 2019 | Dynamic change of cognitive reserve: associations with changes in brain, cognition, and diagnosis | MRI unreported |
| Shen et al., 2019 | Sex differences in the association of APOE ε4 genotype with longitudinal hippocampal atrophy in cognitively normal older people | MRI unreported |
| Firth et al., 2019 | Longitudinal neuroanatomical and cognitive progression of posterior cortical atrophy | Lack of aged control |
| Giudici et al., 2019 | Body weight variation patterns as predictors of cognitive decline over a five year follow-up among community dwelling elderly (MAPT Study) | Lack of aged control |
| Tomadesso et al., 2019 | Is there a specific memory signature associated with Aβ-PET positivity in patients with amnestic mild cognitive impairment? | Not longitudinal > 1 year |
| Madusanka et al., 2019 | One-year follow-up study of hippocampal subfield atrophy in Alzheimer's disease and normal aging | MRI not 1.5T or 3T |
| Brickman et al., 2018 | An MRI measure of degenerative and cerebrovascular pathology in Alzheimer disease | Mixed cohort |
| Zhang et al., 2018 | Cerebrospinal fluid synaptosomal-associated protein 25 is a key player in synaptic degeneration in mild cognitive impairment and Alzheimer's disease | HP atrophy not primary outcome |
| Poulakis et al., 2018 | Heterogeneous patterns of brain atrophy in Alzheimer's disease | Not longitudinal > 1 year |
| Adler et al., 2018 | Characterizing the human hippocampus in aging and Alzheimer's disease using a computational atlas derived from ex vivo MRI and histology | Not longitudinal > 1 year |
| Burke et al., 2018 | Relationship between cognitive performance and measures of neurodegeneration among Hispanic and White Non-Hispanic individuals with normal cognition, Mild Cognitive Impairment, and dementia | Not longitudinal > 1 year |
| Liang et al., 2018 | The role of MRI biomarkers and their interactions with cognitive status and APOE ε4 in nondemented elderly subjects | Not longitudinal > 1 year |
| Ferreira et al., 2017 | The interactive effect of demographic and clinical factors on hippocampal volume: A multicohort study on 1958 cognitively normal individuals | Lack of aged control |
| Schreiber et al., 2017 | Alzheimer disease signature neurodegeneration and APOE genotype in Mild Cognitive Impairment with suspected non-Alzheimer disease pathophysiology | HP atrophy not primary outcome |
| Ramos Bernardes da Silva Filho et al., 2017 | Neurodegeneration profile of Alzheimer's patients: A brain morphometry study | Not longitudinal > 1 year |
| Loewenstein et al., 2017 | Recovery from proactive semantic interference in mild cognitive impairment and normal aging: Relationship to atrophy in brain regions vulnerable to Alzheimer's disease | Not longitudinal > 1 year |
| Wolk et al., 2017 | Medial temporal lobe subregional morphometry using high resolution MRI in Alzheimer's disease | Not longitudinal > 1 year |
| Chetelat et al., 2016 | Atrophy, hypometabolism and clinical trajectories in patients with amyloid-negative Alzheimer's disease | Not longitudinal > 1 year |
| Moretti et al., 2016 | Electroencephalography-driven approach to prodromal Alzheimer's disease diagnosis: from biomarker integration to network-level comprehension | Not longitudinal > 1 year |
| Delli Pizzi et al., 2016 | Atrophy of hippocampal subfields and adjacent extrahippocampal structures in dementia with Lewy bodies and Alzheimer's disease | Not longitudinal > 1 year |
| Teipel et al., 2016 | Does posterior cingulate hypometabolism result from disconnection or local pathology across preclinical and clinical stages of Alzheimer's disease? | Not longitudinal > 1 year |
| Krumm et al., 2016 | Cortical thinning of parahippocampal subregions in very early Alzheimer's disease | Not longitudinal > 1 year |
| Byun et al., 2015 | Heterogeneity of regional brain atrophy patterns associated with distinct Progression rates in Alzheimer's disease | Not longitudinal > 1 year |
| Ossenkoppele et al., 2015 | Atrophy patterns in early clinical stages across distinct phenotypes of Alzheimer's disease | Not longitudinal > 1 year |
| Gispert et al., 2015 | Nonlinear cerebral atrophy patterns across the Alzheimer's disease continuum: impact of APOE4 genotype | Not longitudinal > 1 year |
| Ong et al., 2015 | Aβ imaging with 18F-florbetaben in prodromal Alzheimer's disease: a prospective outcome study | MRI unreported |
| Lim et al., 2015 | Relationships between performance on the Cogstate Brief Battery, neurodegeneration, and Aβ accumulation in cognitively normal older adults and adults with MCI. | MRI unreported |
| Tang et al., 2015 | APOE affects thevolume and shape of the amygdala and the hippocampus in mild cognitive impairment and Alzheimer's disease: Age matters | Not longitudinal > 1 year |
| Lorenzi et al., 2015 | Disentangling normal aging from Alzheimer's disease in structural magnetic resonance images | MRI unreported |
| Gifford et al., 2015 | Subjective memory complaint only relates to verbal episodic memory performance in mild cognitive impairment | Not longitudinal > 1 year |
| Eskildsen et al., 2015 | Structural imaging biomarkers of Alzheimer's disease: predicting disease progression | Not longitudinal > 1 year |
| Cavedo et al., 2014 | Medial temporal atrophy in early and late-onset Alzheimer's disease | Not longitudinal > 1 year |
| Teipel et al., 2014 | Cholinergic basal forebrain atrophy predicts amyloid burden in Alzheimer's disease | Not longitudinal > 1 year |
| Ye et al., 2014 | Hippocampal and cortical atrophy in amyloid-negative mild cognitive impairments: comparison with amyloid-positive mild cognitive impairment | Not longitudinal > 1 year |
| Kilimann et al., 2014 | Subregional basal forebrain atrophy in Alzheimer's disease: a multicenter study | Not longitudinal > 1 year |
| Kerchner et al., 2013 | Shared vulnerability of two synaptically-connected medial temporal lobe areas to age and cognitive decline: a seven tesla magnetic resonance imaging study | Not longitudinal > 1 year |
| Li et al., 2013 | Discriminative analysis of mild Alzheimer's disease and normal aging using volume of hippocampal subfields and hippocampal mean diffusivity: an in vivo magnetic resonance imaging study | Not longitudinal > 1 year |
| Moller et al., 2013 | Different patterns of gray matter atrophy in early- and late-onset Alzheimer's disease | Not longitudinal > 1 year |
| Prestia et al., 2013 | The in vivo topography of cortical changes in healthy aging and prodromal Alzheimer's disease | Not longitudinal > 1 year |
| Whitwell et al., 2012 | Neuroimaging correlates of pathologically defined subtypes of Alzheimer's disease: a case-control study | Not longitudinal > 1 year |
| Shima et al., 2012 | Posterior cingulate atrophy and metabolic decline in early stage Alzheimer's disease | Lack of aged control |
| Devanand et al., 2012 | MRI hippocampal and entorhinal cortex mapping in predicting conversion to Alzheimer's disease | Not longitudinal > 1 year |
| Tondelli et al., 2012 | Structural MRI changes detectable up to ten years before clinical Alzheimer's disease | MRI not 1.5T or 3T |
| Thambisetty, 2012 | Plasma clusterin concentration is associated with longitudinal brain atrophy in mild cognitive impairment. | Lack of aged control |
| Miettinen et al., 2011 | Structure and function of medial temporal and posteromedial cortices in early Alzheimer's disease | Not longitudinal > 1 year |
| Becker et al., 2011 | Amyloid-β associated cortical thinning in clinically normal elderly | Not longitudinal > 1 year |
| Salat et al., 2011 | Hippocampal degeneration is associated with temporal and limbic gray matter/white matter tissue contrast in Alzheimer's disease | Not longitudinal > 1 year |
| Dawe et al., 2011 | Neuropathologic correlates of hippocampal atrophy in the elderly: a clinical, pathologic, postmortem MRI study | Not longitudinal > 1 year |
| Gao et al., 2011 | Complexity of MRI white matter hyperintensity assessments in relation to cognition in aging and dementia from the Sunnybrook Dementia Study | Not longitudinal > 1 year |
| Echavarri et al., 2011 | Atrophy in the parahippocampal gyrus as an early biomarker of Alzheimer's disease | Not longitudinal > 1 year |
| Mueller et al., 2010 | Hippocampal atrophy patterns in mild cognitive impairment and Alzheimer's disease. | Not longitudinal > 1 year |
| Johnson et al., 2010 | Diagnostic utility of cerebral white matter integrity in early Alzheimer's disease | Not longitudinal > 1 year |
| Apostolova et al., 2010 | 3D PIB and CSF biomarker associations with hippocampal atrophy in ADNI subjects | Not longitudinal > 1 year |
| Choo et al., 2010 | Posterior cingulate cortex atrophy and regional cingulum disruption in mild cognitive impairment and Alzheimer's disease | Not longitudinal > 1 year |
| Caroli et al., 2010 | Functional compensation in incipient Alzheimer's disease | Not longitudinal > 1 year |
| Pengas et al., 2010 | Focal posterior cingulate atrophy in incipient Alzheimer's disease | Not longitudinal > 1 year |
| Mormino et al., 2009 | Episodic memory loss is related to hippocampal-mediated beta-amyloid deposition in elderly subjects | Not longitudinal > 1 year |
| Bai et al., 2009 | Absent gender differences of hippocampal atrophy in amnestic type mild cognitive impairment | Not longitudinal > 1 year |
| Frisoni et al., 2008 | Mapping local hippocampal changes in Alzheimer's disease and normal ageing with MRI at 3 Tesla | Not longitudinal > 1 year |
| Chetelat et al., 2008 | Three-dimensional surface mapping of hippocampal atrophy progression from MCI to AD and over normal aging as assessed using voxel-based morphometry | MRI unreported |
| Jagust et al., 2008 | Neuropathological basis of magnetic resonance images in aging and dementia | Average age unreported or < 65 years |
| Hamalainen et al., 2007 | Increased fMRI responses during encoding in mild cognitive impairment | Not longitudinal > 1 year |
| Scher et al., 2007 | Hippocampal shape analysis in Alzheimer's disease: a population-based study | Not longitudinal > 1 year |
| Johnson et al., 2006 | Activation of brain regions vulnerable to Alzheimer's disease: the effect of mild cognitive impairment | Not longitudinal > 1 year |
| Shiino et al., 2006 | Four subgroups of Alzheimer's disease based on patterns of atrophy using VBM and a unique pattern for early onset disease | Not longitudinal > 1 year |
| Xie et al., 2005 | Evaluation of bilateral cingulum with tractography in patients with Alzheimer's disease | Not longitudinal > 1 year |
| Du et al., 2005 | White matter lesions are associated with cortical atrophy more than entorhinal and hippocampal atrophy | Not longitudinal > 1 year |
| deToledo-Morrell et al., 2004 | MRI-derived entorhinal volume is a good predictor of conversion from MCI to AD. | Lack of aged control |
| Pennanen et al., 2004 | Hippocampus and entorhinal cortex in mild cognitive impairment and early AD | Not longitudinal > 1 year |
| Mortimer et al., 2004 | Delayed recall, hippocampal volume and Alzheimer neuropathology: findings from the Nun Study | Not longitudinal > 1 year |
| Meguro et al., 2003 | Corpus callosum atrophy, white matter lesions, and frontal executive dysfunction in normal aging and Alzheimer's disease | Not longitudinal > 1 year |
| Teipel et al., 2003 | Regional pattern of hippocampus and corpus callosum atrophy in Alzheimer's disease in relation to dementia severity: evidence for early neocortical degeneration | Not longitudinal > 1 year |
| Chetelat et al., 2002 | Mapping gray matter loss with voxel-based morphometry in mild cognitive impairment | Not longitudinal > 1 year |
| Jack et al., 2002 | Antemortem MRI findings correlate with hippocampal neuropathology in typical aging and dementia | Not longitudinal > 1 year |
| Dickerson et al., 2001 | MRI-derived entorhinal and hippocampal atrophy in incipient and very mild Alzheimer's disease | Not longitudinal > 1 year |
| Wolf et al., 2001 | Hippocampal volume discriminates between normal cognition; questionable and mild dementia in the elderly. | Not longitudinal > 1 year |
| Convit et al., 2000 | Atrophy of the medial occipitotemporal, inferior, and middle temporal gyri in non-demented elderly predict decline to Alzheimer's disease | Not longitudinal > 1 year |
| Jack et al., 1999 | Prediction of AD with MRI-based hippocampal volume in mild cognitive impairment | Lack of aged control |
| Smith et al., 1999 | MRI temporal lobe volume measures and neuropsychologic function in Alzheimer's disease | Not longitudinal > 1 year |
| Krasuski et al., 1998 | Volumes of medial temporal lobe structures in patients with Alzheimer's disease and mild cognitive impairment (and in healthy controls). | Not longitudinal > 1 year |
| deToledo-Morrell et al., 1997 | Alzheimer's disease: in vivo detection of differential vulnerability of brain regions | Not longitudinal > 1 year |
| Jack et al., 1997 | Medial temporal atrophy on MRI in normal aging and very mild Alzheimer's disease. | Not longitudinal > 1 year |
| Mori et al., 1997 | Medial temporal structures relate to memory impairment in Alzheimer's disease: an MRI volumetric study | Lack of aged control |
| de Leon et al., 1997 | Frequency of hippocampal formation atrophy in normal aging and Alzheimer's disease | Not longitudinal > 1 year |
| de Leon et al., 1996 | In vivo structural studies of the hippocampus in normal aging and in incipient Alzheimer's disease | Not primary literature |
| Frisoni et al., 1996 | Usefulness of simple measures of temporal lobe atrophy in probable Alzheimer's disease | Not longitudinal > 1 year |
| Convit et al., 1993 | Hippocampal atrophy in early Alzheimer's disease: anatomic specificity and validation | Not longitudinal > 1 year |
| Schmidt et al., 1992 | Comparison of magnetic resonance imaging in Alzheimer's disease, vascular dementia and normal aging | Not longitudinal > 1 year |
| Seab et al., 1988 | Quantitative NMR measurements of hippocampal atrophy in Alzheimer's disease | Not longitudinal > 1 year |
| Tabatabaei-Jafari et al., 2019 | Regional brain atrophy predicts time to conversion to Alzheimer's disease, dependent on baseline volume. | Lack of aged control |
| Platero et al., 2019 | Discriminating Alzheimer's disease progression using a new hippocampal marker from T1-weighted MRI: The local surface roughness | Not longitudinal > 1 year |

MRI, magnetic resonance imaging.

## Supplemental Table S3. Data extraction

| Study | Scan Interval | Methods & Type of MRI | Participants | | | Primary Outcome(s) Measured | Results | | Limitations/Strength of Evidence | Eligibility |
| --- | --- | --- | --- | --- | --- | --- | --- | --- | --- | --- |
|  |  |  | Cohort or Recruitment | Total N & n/Group | *Baseline Age & Gender |  | Final Sample Size | Summary Data |  |  |
| Liu et al., 2021 | Baseline, with average of 2.2 years to last scan | Examination of the spatial correlation map of hippocampal subfield atrophy.  3D T-1-weighted scans-   - ADNI-1 = 1.5T MRI - ADNI-2 & ADNI-GO = 3T MRI   Each subject had 1-12 scans for a total of 6525 scans  FreeSurfer longitudinal analysis pipeline was used to delineate hippocampal subfields  Results reported as % atrophy rate of HP subfield volume ratio per year  CSF biomarkers were available for n = 972 subjects, plus cognitive data at all available timepoints. | ADNI-1, ADNI-2, ADNI-GO databases | N Total = 1523   - HC, n = 421 - Stable-MCI, n = 557 - Converted to AD-MCI, n = 241 - AD, n = 304 | N Total = 73.8; M/F = 825/698   - HC = 740; M/F = 199/222 - Stable-MCI = 73.0; M/F = 321/236 - Converted to AD-MCI = 73.8; M/F = 142/99   AD = 75.0; M/F = 163/141 | 1. The atrophy rate of HP subfields in cognitively stable normal individuals, patients with stable MCI, patients who converted from MCI to AD, and patients with AD 2. To what extent atrophy rate of HP subfields predicts the onset age of AD 3. To what extent atrophy rate of HP subfields is associated with AD-related pathology 4. To what extent atrophy rate of HP subfields is correlated with cognitive decline | Not applicable since study used existing data | Adjusted for gender and education  Almost all HP subfields showed an increase in atrophy rate: HC < stable-MCI < converted-MCI < AD (values not provided  Atrophy rate of whole HP was greater in stable-MCI compared to control and converted-MCI compared to stable-MCI and comparable between converted-MCI and AD  Atrophy was selectively associated with declines in certain cognitive domains.  Presubiculm atrophy in converted-MCI patients was associated with CSF tau and corresponded with age on AD onset | - Observational study, using existing data - ADNI subjects are volunteers and from clinics, with good general health - Not all subjects had multiple MRI scans - Mix of 1.5 and 3T MRI | Yes |
| Tabatabaei-Jafari et al., 2019 |  |  |  |  |  |  |  |  |  | No – Study does not include aged HC group |
| Platero et al, 2019 |  |  |  |  |  |  |  |  |  | No – Only MCI patients followed longitudinally |
| Fiford et al., 2019 | Imaging data from all available ADNI-1 timepoints up to 36 months (0-, 6-, 12-, 18-, 24-, and 36-month scans) | Examination of variation in progressive atrophy patterns with age, with a total of 2972 images  Proton density, T1-, and T2-weighted scans   - ADNI-1 = 1.5T MRI   Results reported as HP volume atrophy mL/year  Amyloid pathology confirmed from CSF data | ADNI-1 database | N Total = 683   - Control, n = 191 - MCI, n = 339 - AD, n = 153 | - Control = 75.9; M/F = 51.8%/48.2% - MCI = 75.0; M/F = 62.5%/47.5% - AD = 75.0; M/F = 54.2%/45.8% | 1. The effect of age on atrophy rate patterns in MCI and AD 2. To determine whether any differences in age remained in individuals with confirmed AD pathology (CSF) | Not applicable since study used existing data | Greater age at baseline was associated with significantly increased HP atrophy rate. The increase in HP atrophy rate in controls was equivalent to an acceleration of 50% for a decade increase in age, for someone of average age at baseline (75 years).  In contrast to controls, younger age was associated with greater HP atrophy rate in AD and MCI patients.  AD patients had the highest atrophy rates of 14mL/year for the whole brain and 0.2 mL/year for HP, MCI followed with rates of 10 mL/year for the whole brain and o.1 mL/year for the HP, for APOE negative individuals. Controls had an average atrophy rate of 6 mL/year for the whole brain and 0.06 mL/year for the HP, for APOE negative individuals. | - Observational study, using existing data - ADNI subjects are volunteers and from clinics, with good general health - Methodological limitations | Yes |
| Reas et al., 2018 | Baseline and 2-year follow-up | Examination of brain microstructure using restriction spectrum imaging MRI  3D T1-weight scans   - 3T MRI   Underwent clinical evaluation and cognitive assessment at baseline and follow-up | University of California, San Diego Shiley-Marcos Alzheimer’s Disease Research Center and from the community | N total = 50   - Health control, n = 29 - Impaired, n = 21 | - Health control = 75.4; M/F = 31%/69% - Impaired = 77.5; M/F = 71%/29% | 1. To evaluate whether baseline measures of brain microstructure predict cognitive change and whether change in microstructure over time correlates with change in cognitive function. 2. To characterize changes in aging and early AD. 3. To examine how microstructural changes are associated with normal cognitive aging among individuals without evidence of cognitive impairment. | Follow-up MRI completed by n = 26 HCs, n = 10 MCI, and n = 4 AD | Baseline HP measures predicted cognitive decline and change in HP measures correlated with cognitive decline.  Specifically, in HCsls, higher baseline hippocampal cingulum restricted oriented measures predicted more rapid logical memory immediate recall decline. | - Individuals with MCI and AD were combined into an “impaired” group for analysis - Small sample, with loss of some participants to follow-up - Unequal proportions of men and women | Yes |
| Insel et al., 2015 | Up to 4-year follow-up of MRI data | Examination of relationship between CSF Aß and brain atrophy   - Up to four years of repeated measures of 47 regional volumes of grey matter were averaged over right and left hemispheres. - ADNI-1 = 1.5T MRI   Results reported atrophy rate mm^3^/year  At baseline, CSF sample was collected by lumbar puncture and Aß42 measured. | ADNI-1 database | N Total = 291   - Control, n = 108 - MCI, n = 183 | N Total = 74.8; M/F = 61%/39%   - Control = 75.5; M/F = 52%/48% - MCI = 74.4; M/F = 66%/34% | Estimation of the association between CSF Aß42 and brain atrophy in each of the 47 regions, including HP | Not applicable since study used existing data | In MCI, periods of atrophy acceleration are evident in several brain regions, including HP over the course of CSF Aß accumulation.  Although a high number of control subjects were beyond the clinical threshold for Aß positivity, excess accumulation did not necessarily result in an immediate increase in atrophy rate. | - Observational study, using existing data - ADNI subjects are volunteers and from clinics, with good general health - Not all subjects had multiple MRI scans up to 4 years - Limited Aß42 data | Yes |
| Tarawneh et al., 2015 | Follow-up of 2 to 3 years (mean duration = 2.7 years) | Examination of CSF biomarkers in predicting brain atrophy  Structural MRI   - 3T or 1.5T MRI, results reported as volume mm^3^   n = 192 participants underwent MRI within 1.1 years of baseline CSF biomarker measurements (lumbar puncture) | Charles F. and Joanne Knight Alzheimer’s Disease Research Center, Washing University School of Medicine | N Total = 87   - Cognitively NC, n = 64 - AD, n = 23 | N Total = 72.6 (at lumbar puncture)   - Cognitively NC = 72.3; M/F =19/45 - AD = 73.6; M/F = 9/14 | Correlation between baseline CSF biomarker measures (VILIP-1, tau, p-tau 181, Aß42) atrophy over the follow-up period | Follow-up completed by all, mean duration of follow-up 2.7 years (0.9-7.9 years) | In AD, baseline levels of VILIP-1, tau, p-tau predicted whole brain and HP atrophy.  Cognitively NCs with CSF markers in the upper tercile had higher rates of whole brain and HP atrophy compared to those with lower levels (adjusting for age, sex, imaging system type, and APOE4 genotype).  CSF biomarker levels and rates of whole-brain and regional atrophy in this subset of controls were similar to those with AD.  The mean (SE) adjusted rate of atrophy in control cohort was 0.003 (0.001) points per year to normalized whole-brain volume (-0.4% annual change from baseline) -100 (25) mm^3^ per year for HP volume (-1,3% annual change from baseline) and 0.05% (0.02) mm per year for entorhinal thickness (-1.3% annual change from baseline. | - Relatively short follow-up and varied years of follow-up - Small sample size - Mix of 3T and 1.5T for structural MRI | Yes |
| Lo et al., 2011 | After the baseline visit, visits occurred at 6- or 12-month intervals. Control or MCI subjects were followed up for 3 years, whereas those with AD were followed up for 2 years at maximum. | Examination of the trajectories of Aβ42 level in CSF, FDG uptake using PET, and HP volume using MRI and their relative associations with cognitive change at different stages in aging and Alzheimer disease  T1-weighted scans-  • 1.5T MRI, reported as HP volume, mm³/month | ADNI database | N Total = 819  • NC, n = 229  • MCI, n = 397  • AD, n = 193 | • NC = 75.1; M/F = 119/110 • MCI = 74.0; M/F = 256/141 • AD = 74.6; M/F = 102/91 | Rates of change in level of Aβ42 in CSF, FDG uptake, HP volume, and the Alzheimer Disease’s Assessment Scale- cognitive subscale score during up to 36 months of follow-up | Not applicable since study used existing data | The rate of MRI HP atrophy was significantly slower in participants with NC (-2.95 mm³/month) than in participants with MCI (-5.52 mm³/month) or AD (-8.01 mm³/month) and slower in participants with MCI than in participants with AD.  Positive APOE4 accelerated HP atrophy in the MCI and AD groups. | • Observational study, using existing data  • ADNI subjects are volunteers and from clinics, with good general health  • The number of repeated measures available for longitudinal analysis varied across different biomarkers and diagnostic groups | Yes |
| Li et al., 2011 | Varied, one or more years later- Each subject was scanned on two or more visits. All scan intervals were rounded to the nearest year (e.g., a scan occurring 6 months or more from initial scan would be classified as year 1) | Examination of gray matter changes in AD progression  T1-weighted scans- • 1.5T MRI, reported as annual rate of atrophy | OASIS | N Total = 150 • Normal aging, n = 72 • AD, n = 64, plus n = 14 subjects converted from normal aging during the study | • Normal aging = 71.8; M/F = 19/53 • AD = 77.0; M/F = 40/38 | A) To determine the pattern of neuropathological expansion in AD and which regions show earliest disease effects B) To determine whether the atrophy rates in AD are similar or different across affected regions C) To determine whether patterns of atrophy across affected regions are inter-dependent or independent | Not applicable since study used existing data | A) The initial gray matter deficits was generally bilateral, located in anterior regions of the HP and entorhinal cortex. By time point 2, there was a progression of atrophy in the medial temporal regions. HP gray matter loss extended so that most of the structure was affected.  B) There was variation across the AD subjects in terms of yearly rate of gray matter atrophy for both HP and MTG, but it was typically 1% for HP and 0.5% for MTG, which was two times and five times greater than atrophy rate in controls. C) Results indicated that for both the HP and MTG, local changes in gray matter concentration were the best predictors of future changes | • Observational study, using existing data • The time interval between first and second time points was not the same for all the subjects • Combined data from AD subjects and converter subjects | Yes |
| Skup et al., 2011 | Individuals in the AD group contributed (on average) 3.03 scans over a 2-year period, individuals in the aMCI group contributed 3.71 scans over a 3-year period, and HCs contributed 3.60 scans over a 3-year period | Examination of sex differences in gray matter atrophy patterns, brain volume of various structures in the brain's grey matter were measured using serial MRI  3D T1-weighted scans- • 1.5T MRI | ADNI database | N Total = 687 • HC, n = 224  • aMCI, n = 266 • Probable AD, n = 197 | • HC = 76.00; M/F = 114/110  • aMCI = 74.91; M/F = 176/90 • Probable AD = 75.65; M/F = 101/96 | To determine differences in patterns of volume change over time by sex and diagnostic group | Not applicable since study used existing data | Validation findings showed that AD and aMCI patients had decreased HP volume over time compared to controls.  Males and females in the AD and aMCI groups showed different patterns of decline over time compared to controls in the bilateral precuneus, bilateral caudate nucleus, right entorhinal gyrus, bilateral thalamus, bilateral middle temporal gyrus, left insula, and right amygdala. | • Observational study, using existing data • ADNI subjects are volunteers and from clinics, with good general health • Methodological limitations | Yes |
| Wang et al., 2009 | Baseline and up to 2-years; 12-month intervals | Examination of the annual change rates in cognitive performance and HP volume.  T2-weighted scans- • 1.5T MRI, reported as percent annual atrophy rate  All participants received an annual neuropsychological assessment with the Cognitive Abilities Screening Instrument.  All patients were categorized by the presence or absence of the APOE4 allele (i.e., E4+ or E4-). | Subjects were recruited from neurological clinics and the study was conducted at Taipei Veterans General Hospital. | N Total = 78 • NC, n = 20 • Stable MCI, n = 39 • Progressive MCI, n = 19 | • NC = 75.1; M/F = 11/9 • stable MCI = 75.6; M/F = 31/8 • progressive MCI = 77.6; M/F = 12/7 | 1) To evaluate differences in annual change rates in neuropsychological and MRI volumetric assessments  2) To determine whether APOE4 contributes to a higher rate of HP atrophy in normal aging and MCI subjects 3) To assess the correlation between HP volume and neuropsychological test scores. | Follow-up completed by all, mean duration of follow-up 22.5 months (10.7–34.8 months) | Compared with the control group, progressive MCI subjects had significantly greater atrophy rates in both right and left HP.   Compared to the stable MCI group, the progressive MCI group had a faster atrophy rate in right, but not in left, HP.   There were no APOE4 group effects on neuropsychological or MRI volumetric assessments.  In the MCI groups, there was a significant correlation between the decline in memory test scores and HP atrophy rates. | • Small sample size and more males than females  • Variable follow-up period | Yes |
| Whitewell et al., 2007 |  | The median time interval between the first MRI and conversion to AD was 3 years (range 1.7–4.2 years), and the median interval between the second MRI and conversion was 1 year (range 0.8–1.5 years). | Examination of the progression of cerebral atrophy over serial MRI: first scan approximately three years before conversion, the second approximately one year before conversion, and the third at the time of conversion  3D T1-weighted scans- • 1.5T MRI   APOE testing was also performed | Mayo Clinic ADRC and ADPR | N Total = 66 • NC, n = 33 • aMCI converters to AD, n = 33 | NC, median = 78; M/F = 14/19 • aMCI converters to AD, median = 78; M/F = 14/19 | Not applicable since study used existing data | The pattern of grey matter loss in aMCI three years before conversion was focused primarily on the medial temporal lobes, including HP. The extent and magnitude of the cerebral atrophy further progressed by the time the subjects were one year before conversion and extended to include the entire extent of HP By the time the subjects had a clinical diagnosis of AD the pattern of grey matter atrophy had become still more widespread.  The HP showed progressive atrophy throughout the disease course, with the severity of HP loss detected on MRI increasing at each time-point. | • Observational study, using existing data • Small sample size • Variable follow-up period | Yes |
| Jack et al., 2005 | The mean interval between the two MRI exams was 1.4 years (range, 0.9 to 2.0 years) in normal subjects and 1.3 years (range, 0.7 to 2.6 years) in MCI subjects. | Atrophy rates of four different brain structures were measured from a pair of MRIs separated by one to two years.   3D T1-weighted scans- • 1.5T MRI  The time of the second scan marked the beginning of the clinical observation period. | Mayo Clinic ADRC and ADPR | N Total = 163 • NC, n = 91 • MCI, n = 72   NC converted to MCI (n = 11) or AD n = (2), MCIs converted to AD | Age at 2nd scan: • NC = 81.9; M/F = 36/55 • MCI = 80.0; M/F = 41/31 | To determine whether atrophy rate measured is associated with time to subsequent clinical conversion to a more impaired state among cognitively normal elderly subjects and subjects with aMCI | Not applicable since study used existing data | Rates of atrophy for the MCI cohort were greater that those of the NCs for all four measures, including HP (-1.7 vs. -3.3 annual % change).   During follow-up, n = 39 MCI subjects converted to AD while n = 13 control subjects converted to MCI (11) or AD (2). | • Observational study, using existing data • Relatively short follow-up and varied years of follow-up | Yes |
| Silbert et al., 2003 | Followed for an average of 5.8 years to autopsy; ~28 months from last MRI to death | Examination of whether changes in antemortem MRI brain volume measurements are predictors of subsequent AD pathology • 1.5T MRI  Image analysis of regional volumes was performed using REGION.  The median postmortem interval was 12.3 hours (range 1.5 to 48 hours) | Oregon Aging and AD Center at Oregon Health and Science University | N Total = 39 • NC, n = 15 • Cognitive impairment, n = 24 | N Total = 84.00; M/F = 54%/46% • NC = 87.90; M/F = 53.3%/46.7% • Cognitive impairment = 81.53; M/F = 54.2%/45.8% | A) To confirm that the degree of brain volume atrophy observed prior to death assessed by MRI volumetrics is predictive of subsequent AD; B) To determine which specific brain regions were best able to predict AD pathology on the basis of volume measurement; C) To determine whether volume measurement change obtained over time was more sensitive to the pathologic changes of AD than the last brain volume measured prior to death | All subjects followed until death | Results indicated that in subjects with cognitive impairment, there was a relationship between the degree of HP neurofibrillary tangle burden and total HP volume prior to death. In the cognitively impaired subjects, no such relationship existed between the amount of neurofibrillary tangle and the rate of HP volume change.   In control subjects, no significant relationship was found between HP neurofibrillary tangle burden and last HP volume prior to death or the rate of HP volume change over time. | • Observational study, using existing data • Not all subjects had multiple MRI scans • Small sample • 2/39 subjects had mixed AD and vascular pathology | Yes |

3D, three-dimensional; Aβ, amyloid-β (+, -); AD, Alzheimer’s disease; ADNI, Alzheimer’s Disease Neuro-Imaging Initiative; ADPR, Alzheimer’s Disease Patient Registry; ADRC, Alzheimer’s Disease Research Centre; APOE, apolipoprotein E; aMCI, amnestic mild cognitive impairment; CSF, cerebral spinal fluid; FDG; fludeoxyglucose F18; HC, healthy control; HP, hippocampus; M, male; MCI, mild cognitive impairment; MRI, magnetic resonance imaging; MTG, medial temporal gyrus; NC, normal control; OASIS, Open Access Structural Imaging Series; p-tau, phosphorylated tau; SE, standard error; VILIP-1, visinin-like protein 1.
